# Results of two consecutive treatment protocols in Polish children with acute lymphoblastic leukemia

**DOI:** 10.1038/s41598-020-75860-6

**Published:** 2020-11-19

**Authors:** Joanna Zawitkowska, Monika Lejman, Michał Romiszewski, Michał Matysiak, Magdalena Ćwiklińska, Walentyna Balwierz, Joanna Owoc-Lempach, Bernarda Kazanowska, Katarzyna Derwich, Jacek Wachowiak, Maciej Niedźwiecki, Elżbieta Adamkiewicz-Drożyńska, Joanna Trelińska, Wojciech Młynarski, Andrzej Kołtan, Mariusz Wysocki, Renata Tomaszewska, Tomasz Szczepański, Marcin Płonowski, Maryna Krawczuk-Rybak, Justyna Urbańska-Rakus, Katarzyna Machnik, Tomasz Ociepa, Tomasz Urasiński, Agnieszka Mizia-Malarz, Grażyna Sobol-Milejska, Grażyna Karolczyk, Jerzy Kowalczyk

**Affiliations:** 1 Department of Pediatric Hematology, Oncology and Transplantology, Medical University of Lublin, Antoni Gębala Street 6, 20-093 Lublin, Poland; 2 Laboratory of Genetic Diagnostics, Medical University of Lublin, Lublin, Poland; 3 Department of Hematology and Pediatrics, Children’s Hospital, Warsaw, Poland; 4 Department of Pediatric Oncology and Hematology, Children’s University Hospital, Jagiellonian University, Kraków, Poland; 5 Department of Pediatric Transplantology, Oncology, Hematology, Medical University of Wrocław, Wrocław, Poland; 6 Department of Pediatric Oncology, Hematology and Transplantology, Medical University of Poznań, Poznań, Poland; 7 Department of Pediatrics, Hematology, Oncology and Endocrinology, Medical University of Gdańsk, Gdańsk, Poland; 8 Department of Pediatrics, Oncology and Hematology, Medical University of Łódź, Łódź, Poland; 9 Department of Pediatrics, Hematology and Oncology, Collegium Medicum of Bydgoszcz, Bydgoszcz, Poland; 10 Department of Pediatrics, Hematology and Oncology, Medical University of Zabrze, Zabrze, Poland; 11 Department of Pediatric Oncology, Hematology, Medical University of Białystok, Białystok, Poland; 12 Department of Pediatric Hematology and Oncology, Center of Pediatrics and Oncology, Chorzów, Poland; 13 Department of Pediatrics, Hematology and Oncology, Medical University of Szczecin, Szczecin, Poland; 14 Department of Pediatric Oncology, Hematology and Chemotherapy, Medical University of Katowice, Katowice, Poland; 15 Department of Pediatric Oncology and Hematology, Children’s Hospital, Kielce, Poland

**Keywords:** Cancer, Oncology, Risk factors

## Abstract

The aim of the study was to retrospectively compare the effectiveness of the ALL IC-BFM 2002 and ALL IC-BFM 2009 protocols and the distribution of risk groups by the two protocols after minimal residual disease (MRD) measurement as well as its impact on survival. We reviewed the medical records of 3248 patients aged 1–18 years with newly diagnosed ALL who were treated in 14 hemato-oncological centers between 2002 and 2018 in Poland. The overall survival (OS) of 1872 children with ALL treated with the ALL IC 2002 protocol was 84% after 3 years, whereas the OS of 1376 children with ALL treated with the ALL IC 2009 protocol was 87% (*P* < 0.001). The corresponding event-free survival rates were 82% and 84% (*P* = 0.006). Our study shows that the ALL IC-BFM 2009 protocol improved the results of children with ALL compared to the ALL IC-BFM 2002 protocol in Poland. This analysis confirms that MRD marrow assessment on day 15 of treatment by FCM-MRD is an important predictive factor.

## Introduction

Acute lymphoblastic leukemia (ALL) is the most frequent malignant disorder in the pediatric population, with a peak incidence between 2 and 5 years of age. Currently, the overall survival of children with ALL is more than 80% due to intensive therapy. The main problem is relapse, which occurs in 20% of children with ALL, and the prognosis of these patients is poor^1,2^. Early response to treatment is strongly associated with the risk of recurrence. This is why it is important to establish new prognostic factors that are more sensitive^3^. ALL is the first cancer in which the assessment of early response to therapy by monitoring minimal residual disease (MRD) has been indicated to be an essential tool in making therapeutic decisions^1,4^.

The International Berlin–Frankfurt–Münster Study Group (I-BFM-SG) includes national study groups from over 30 countries around the world that collaborate in working committees to address the essential aspects of childhood leukemia and lymphoma research. The European cooperative groups assessed modifications to basically all elements of therapy in randomized trials^5^. In the nineties, I-BFM-SG research reported that the detection of MRD at two consecutive time points (on days 33 and 78 of therapy) is helpful for distinguishing patients with a good prognosis (standard risk, MRD-SR) from patients with an intermediate prognosis (intermediate risk, MRD-IR) or a poor prognosis (high risk, MRD-HR)^6^. Gradually, the I-BFM group covered new countries with inadequate skills and less experience with intensive chemotherapy schedules, resulting in the study being adapted to local conditions. The ALL-IC BFM 2002 protocol was the first intercontinental randomized clinical trial of the I-BFM-SG and was advised for countries with limited resources for PCR-based MRD assessment. Based on the pioneering findings of the I-BFM-SG in measuring the early peripheral blood response to prednisone on day 8 and bone marrow blasts on day 15, all patients could be categorized into risk groups by available methods. In the next study protocol, ALL-IC BFM 2009, the new stratification based on minimal residual disease (MRD) evaluation was applied. The ALL-IC BFM group agreed to use only flow cytometry (FCM) for MRD analysis for this study, as the utilization of PCR-based methods appeared difficult in most participating countries. One of the main aims of the ALL IC 2009 protocol was to determine if the outcome of patients with SR, according to the ALL IC 2002 criteria for SR and FC-MRD burden < 0.1% at day 15, would be better than the outcome that could be expected from stratification by the criteria of ALL-IC 2002^5^.

The Polish Pediatric Leukemia/Lymphoma Study Group joined the ALL IC-BFM 2002 study in 2002 and then the ALL IC-BFM 2009.

In this study, we report the demographics, incidence and clinical outcomes of children with ALL in the Polish population. The aim of the study was to retrospectively compare the effectiveness of the ALL IC-BFM 2002 and ALL IC-BFM 2009 protocols and the distribution to risk groups by the two protocols after MRD measurement as well as its impact on survival. We were also interested in whether our analysis would confirm high event-free survival in risk groups, especially in the SR-MRD group.

## Results

### Patient characteristics

The demographics of the two groups of patients treated with the ALL IC 2002 (1872 pts) and ALL IC 2009 (1376 pts) protocols are presented in Table 1. There were statistically significant differences between these groups in the following features: average age, WBC count at diagnosis, risk group, BCR/ABL1-positive fusion gene, immunophenotype and BM result on days 15 and 33 of the induction phase. We additionally calculated the effect size for each comparison. Effect size enables us to assess the strength/size of the relationship between variables in each test, independent of sample size. Therefore, based on the weak effect sizes of age, WBC count and immunophenotype, we can conclude that the differences in these variables between the groups, which were statistically significant (< 0.05), were not important from a clinical standpoint.

**Table 1:** Patients’ characteristics treated the ALL IC 2002 and ALL IC 2009 protocols.

Characteristic	ALL IC 2002	ALL IC 2009	*P*
No. of patients	1872	1376	
Sex, *n* (%)			
Female	813 (43.4)	597 (43.4)	> 0.999
Male	1059 (56.6)	779 (56.6)	
Age, years, mean ± SD	6.94 ± 4.64	6.49 ± 4.37	0.005
Age < 6 years, *n* (%)	1031 (55.1)	818 (59.4)	0.014
Age ≥ 6 years, *n* (%)	841 (44.9)	558 (40.6)	
WBC, µl			
< 20,000	1208 (64.5)	1080 (78.5)	< 0.001
≥ 20,000	664 (35.5)	296 (21.5)	
Infiltration CNS, *n* (%)			
Status 1	1663 (88.8)	1196 (86.9)	0.091
Status 2	108 (5.8)	106 (7.7)	
Status 3	101 (5.4)	74 (5.4)	
Organ infiltration, *n* (%)			
Spleen	1033 (55.2)	767 (55.7)	0.778
Liver	1207 (64.5)	867 (63.0)	0.410
Mediastinum	145 (7.8)	74 (5.4)	0.010
Testes	20 (1.1)	12 (0.9)	0.704
Risk group, *n* (%)			
SRG	611 (32.6)	192 (14.0)	< 0.001
IRG	898 (48.0)	868 (63.1)	
HRG	363 (19.4)	316 (23.0)	
Immunophenotype, *n* (%)			
Pre-B			
Common positive	1081 (57.7)	1100 (79.9)	< 0.001
Common negative	578 (30.9)	111 (8.1)	
T-ALL	210 (11.2)	164 (11.9)	
AHL	3 (0.2)	1 (0.1)	
Genetic aberration, *n* (%)			
Karyotype with aberration *BCR/ABL1*	65 (3.5)	19 (1.4)	< 0.001
Karyotype with rearrangement *KMT2A*	32 (1.7)	21 (1.5)	0.773
* KMT2A/AFF1(formerly MLL-AF4)*	5 (0.3)	4 (0.3)	0.999
Hypodiploidy, *n* (%)	22 (1.2)	17 (1.2)	> 0.999
Steroids response, *n* (%)			
Good	1645 (87.9)	1228 (89.2)	0.249
Poor	227 (12.1)	148 (10.8)	
Blast (BM) day 15, *n* (%)			
M1	1319 (70.5)	1028 (74.7)	0.024
M2	368 (19.7)	238 (17.3)	
M3	185 (9.9)	110 (8.0)	
Blast (BM) day 33, *n* (%)			
M1	1810 (96.7)	1356 (98.5)	0.004
M2	40 (2.1)	4 (1.0)	
M3	22 (1.2)	6 (0.4)	
MRD, *n* (%)			
< 0.01	n/a	468 (34.0)	n/a
≥ 0.01 < 10	n/a	710 (51.6)	
> 10	n/a	198 (14.4)	

*CNS* central nervous system, *BM* bone marrow.

The difference between the frequency of *BCR/ABL1* rearrangement (3.5% for ALL IC 2002 and 1.4% for ALL IC 2009) was because some of the children with ALL Ph+ were treated with the EsPhALL (European intergroup study on postinduction treatment of ALL Ph+) protocol, in which imatinib was incorporated into combination chemotherapy regimens from 2012/2013.

There were no significant differences between these groups in the remaining features. Most children were male. Splenomegaly and hepatomegaly were reported in the majority of children (over 50%), and mediastinal involvement from the leukemic cells rarely occurred. The rearrangements of *KMT2A (MLL)* were positive in less than 2% of patients. Most patients had a good response to prednisone on day 8.

### Treatment results

#### The ALL IC 2002 protocol

The date of the last follow‐up was 31 December 2016. The median follow-up time for the entire group was 6.41 years. Details of the treatment results were published by Kowalczyk et al.^7^.

#### The ALL IC 2009 protocol

The date of the last follow‐up was 31 December 2019. The median follow-up time for the entire group was 3.36 years. A total of 88 (14.3%) deaths were noted in the entire group, including 2 (2.3%) deaths in the SR group, 40 (4.5%) in the IR group, and 46 (52.3%) in the HR group. Considering the size of the risk groups, the death rate observed was 1.1% (2/192 pts) in the SR group, 4.5% (38/868 pts) in the IR group, and 15.2% (48/316 pts) in the HR group. The cumulative death risk at 5 years was estimated to be 1.0% (SE 0.007) in the SR group, 6.1% (SE 0.014) in the IR group, and 18% (SE 0.026) in the HR group.

In 14 (16%) cases, deaths happened during induction therapy before complete remission (CR) was achieved, and the estimated death rate during induction therapy was 1.02% (0% for the SR group, 0.65% for the IR group, and 0.37% for the HR group). A total of 31 (35%) deaths were noted during the CR phase (death rate of 2.25%, with 0.15% in the SR group, 0.8% in the IR group, and 1.3% in the HR group). Deaths related to relapse were noted in 40 (45.4%) children.

Relapses were observed in 128 children (9.3%), of which 5 (3.9%) cases were in the SR group, 80 (6.3%) were in the IR group, and 43 (33.6%) were in the HR group. The cumulative relapse risk was 4.6% (SE 0.020) in the SR group, 13.6% (SE 0.016) in the IR group, and 20.8% (SE 0.031) in the HR group.

#### Comparison of the treatment results of both protocols

**Figure 1:**
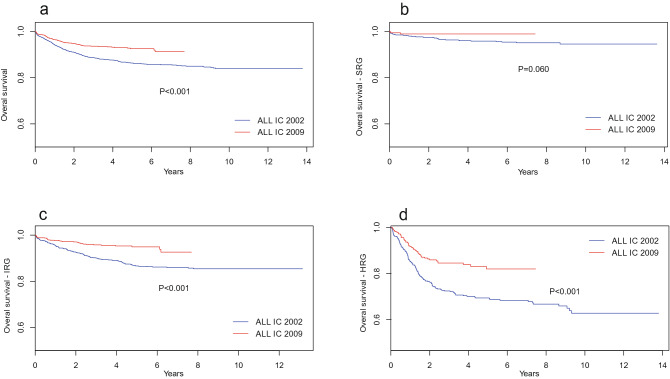
Overall survival for entire cohort of children with ALL (**a**) and by risk groups (**b**–**d**) for two protocols. *SRG* standard risk group, *IRG* intermediate risk group, *HRG* high risk group.

**Figure 2:**
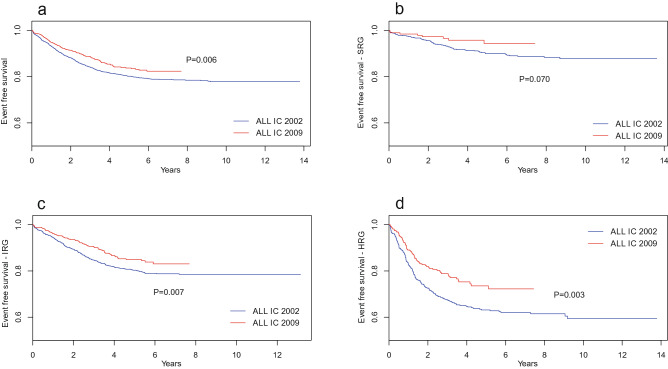
Event-free survival for entire cohort of children with ALL (**a**) and by risk groups (**b**–**d**) for two protocols. *SRG* standard risk group, *IRG* intermediate risk group, *HRG* high risk group.

The overall survival (OS) rates of children with ALL treated with the ALL IC 2002 and ALL IC 2009 protocols were 84% and 87%, respectively, after 3 years in Poland, and the difference was statistically significant (*P* < 0.001); the corresponding event-free survival (EFS) rates of the two analyzed groups were 82% and 84% after 3 years (*P* = 0.006). The OS and EFS of the two groups of children with ALL and by risk groups are presented in Figs. 1 and 2, respectively.

**Figure 3:**
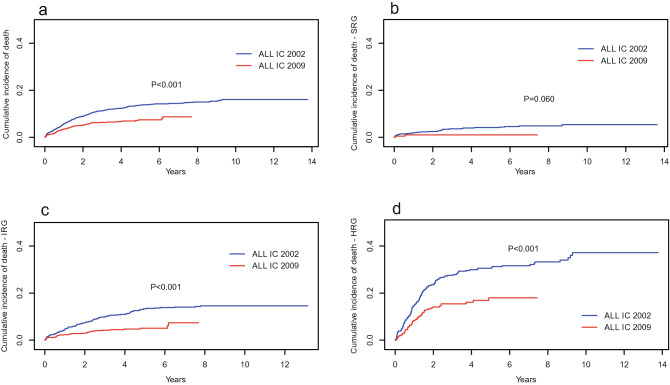
Cumulative incidence of deaths for entire cohort of children with ALL (**a**) and by risk groups (**b**–**d**) for two protocols. *SRG* standard risk group, *IRG* intermediate risk group, *HRG* high risk group.

**Figure 4:**
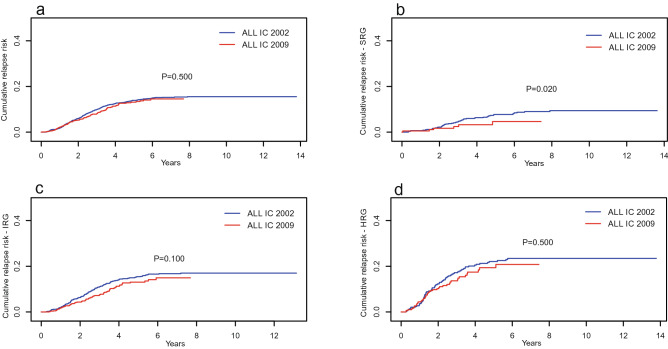
Cumulative incidence of relapse for entire cohort of children with ALL (**a**) and by risk groups (**b**–**d**) for two protocols. *SRG* standard risk group, *IRG* intermediate risk group, *HRG* high risk group.

The cumulative incidence of death (CID) of all groups with the ALL IC 2002 protocol was higher than that with the ALL IC 2009 protocol, and the difference was statistically significant (*P* < 0.001) (Fig. 3a). The CID analyzed by risk group showed that IR and HR patients treated with the ALL IC 2002 protocol had a higher CID than those treated with the ALL IC 2009 protocol (*P* < 0.001) (Fig. 3b–d). The cumulative incidence of relapse (CIR) of the whole group was higher for ALL IC 2002, but there was no significant difference (Fig. 4a). The CIR analyzed by risk groups presented a significant difference only for SR children (*P* = 0.02) (Fig. 4b–d).

## Discussion

This study is a large retrospective analysis comparing the results of treatment with two protocols, ALL IC 2002 and ALL IC 2009, for children with ALL in Poland. In our cohort, the distribution into risk groups (especially to the standard risk group) and response to induction therapy differed significantly in both protocols. FCM-based MRD, which was implemented for risk stratification in the ALL IC 2009 protocol, had a substantial impact, leading to more accurate risk group assignments and induction phase outcome predictions. The level of MRD was only evaluated on day 15 of the induction phase of the protocol.

It is known that MRD is a strong prognostic factor for determining risk groups and subsequently the intensity of post-induction therapy (from significant treatment reduction to mild or strong intensification)^5,8–11^. The technologies of MRD measurement for childhood ALL studies are based on molecular or flow cytometry, and the use of these methods depends on the resources available. In Poland, the RQ-PCR method was not achievable during therapy with the ALL IC 2009 protocol.

The collaboration of two large study groups of childhood ALL, Associazione Italiana di Ematologia Pediatrica and Berlin-Frankfurt-Münster (AIEOP-BFM ALL) 2000, studied the classification of children into standard, intermediate and high-risk groups based on PCR-MRD at the end of induction therapy and at the end of consolidation therapy. AIEOP also analyzed the prognostic effect of MRD as measured by FCM on day 15 of the induction phase^12^.

Basso et al. published the FCM-MRD results of 815 children, aged 1 to younger than 18 years, with newly diagnosed ALL between 2000 and 2004 in the AIEOP-BFM-ALL 2000 study. Bone marrow samples from patients were collected on day 15 of the induction of therapy.

The patients were stratified into three risk groups by FCM: standard (0.1% blast cells; 42% of the total), intermediate (0.1–10%; 47%), sand high (≥ 10%; 11%). The cumulative incidences of relapse after 5 years for the corresponding groups were 7.5% (SE, 1.5), 17.5% (SE, 2.1), and 47.2% (SE, 5.9). In this study, the authors concluded that FCM-MRD measurement and one time point were important prognostic factors^12^.

Flohr et al. reported the results of the multicenter study AIEOP-BFM ALL 2000, in which MRD-PCR-based stratification was feasible in almost 80% of patients. In this study, the patients were stratified by PCR-MRD detection after induction (TP1) and consolidation treatment (TP2). PCR target identification was performed in 3341 patients between 2000 and 2004. MRD-based risk group assignment was feasible in 2594 (78%) patients: 40% were classified as MRD-SR, 8% as MRD-HR (MRD × 10^−3^ at TP2), and 52% as MRD-IR. The remaining 823 patients were stratified according to clinical risk features: HR (n = 108) and IR (n = 715)^6^. Similarly, Conter et al. confirmed that the MRD response detected by PCR at 2 time points is highly predictive for relapse in childhood pB-ALL in the AIEOP-BFM ALL 2000 study^13^. Pui et al. reported 498 children with newly diagnosed ALL who were treated in the St. Jude Total Therapy XV study. This was the first clinical trial in which MRD was determined prospectively during and after remission induction therapy. In this study, MRD was determined in bone marrow specimens by PCR on days 19 and 46 of remission induction and on week 7 of continuation treatment. The authors reported that two of these time points (days 19 and 46) were valuable for the stratification of patients into risk groups^14^.

In our study, we present the results of two ALL treatment regimens in Poland between 2002 and 2018. We observed that the overall survival of children treated with the ALL IC 2009 protocol was significantly improved compared to that of children treated with the ALL IC 2002 protocol, and this was particularly pronounced in IR and HR children. Similarly, the event-free survival rate of patients treated with the ALL IC 2009 protocol was higher than that of patients treated with the ALL IC 2002 protocol. Our analysis confirmed significantly higher event-free survival in the SR-MRD group of patients compared to the SR patients according to the ALL IC 2002 criteria. Regarding the cumulative incidence of death and the cumulative relapse rate of the two analyzed groups of children, we noticed that these rates were higher in children treated with the ALL IC 2002 protocol. The improvement in outcomes could be attributed to the introduction of FCM-based MRD assessment, the redefinition of stratification into risk groups and the development of supportive therapy (novel broad-spectrum antibiotics, antifungal drugs).

The results of the ALL IC 2009 protocol are comparable to those of reported studies conducted by major collaborative groups^3,15^.

Pui et al. presented a review of the impact of collaborative studies (14 study groups) on the heterogeneity and treatment of ALL in children and teenagers between 1995 and 2011. These studies confirmed that MRD assessment is the most predictive indicator in both B-cell and T-cell ALL. The authors reported the clinical outcomes of several therapeutic regimens. The 5-year EFS ranged from 75.9% (AIEOP-95) to 87.3% (NOPHO-2000 and SJCRH XV), and the 5-year OS ranged from 85.4% (CoALL-97) to 93.5% (SJCRH XV)^16–21^.

In conclusion, our study demonstrates that the ALL IC-BFM 2009 protocol improved the clinical outcomes of children with ALL compared to the ALL IC-BFM 2002 protocol in Polish pediatric oncohematology centers. This analysis confirms that the bone marrow assessment on day 15 of treatment (early time point) by FCM-MRD is an important predictive factor. Due to the MRD assessment, many patients were redirected from the SR group to the IR group, resulting in the EFS being higher in the SR group.

## Patients and methods

### Study group

The study protocols were approved by the Ethics Committee of Medical University of Lublin. All procedures performed in studies involving human participants were in accordance with the ethical standards of the institutional and/or national research committee and with the 1964 Helsinki Declaration and its later amendments or comparable ethical standards. Informed consent was obtained from the parents or guardians of the participants until the age of 18.

We reviewed the medical records of 3248 patients aged 1–18 years with newly diagnosed acute lymphoblastic leukemia who were treated in 14 hemato-oncological centers in Poland during two periods, November 2002 to November 2011 (ALL IC 2002) and December 2011 to September 2018 (ALL IC 2009). Infants younger than one year were excluded. Clinical data were collected at the time of diagnosis, including age, organomegaly, central nervous system (CNS) infiltration, white blood cell count (WBC), immunophenotyping, presence of *BCR/ABL1* and *KMT2A* rearrangements and data of response to steroids and bone marrow results on days 15 and 33 of therapy. The demographic details are presented in the Results section in Chromosomal abnormalities were analyzed at diagnosis with the bone marrow (BM) of each patient according to the standard protocols and were examined by conventional cytogenetic (CC) and fluorescence in situ hybridization (FISH) methods. A central review of the results of myelogram, flow cytometry and genetic analysis at the national level was mandatory.

### Definition of remission status

The prednisone response in the peripheral blood (PB) and bone marrow morphological response were evaluated in both protocols. The absolute blast count (ABC) in PB on day 8 after 7 days of prednisone pre-phase and one dose of intrathecal methotrexate on day 1 were evaluated. The prednisone-good responders (PGRs) are patients with an ABC on day 8 of < 1000/μL PB, and the prednisone-poor responders (PPRs) are patients with an ABC on day 8 of ≥ 1000 blasts/μL.

M1 marrow was defined as bone marrow with < 5% blasts, M2 marrow was defined as bone marrow with 5% to 24% blasts, and M3 marrow was defined as bone marrow with ≥ 25% blasts on the 15th and 33rd days of the induction phase of therapy. The definition of relapse, which was followed after the first complete remission, was ≥ 25% blasts in the bone marrow or disease involvement elsewhere.

MRD assessment was only used in the ALL IC 2009 protocol. MRD can be detected by PCR (polymerase chain reaction) or FCM (flow cytometry) methods. Real-time quantitative PCR (RQ-PCR)-based MRD is detected by the polymerase rearrangement of the immunoglobulin and T-cell gene receptor. It presents high analytical sensitivity (< 10^−5^) and is highly standardized, but some patients lack a suitable PCR marker. The methodology is highly complex and costly. At that time, we did not have such specific laboratories in our country, and the determination of PCR-MRD required the development and implementation of research standards for residual disease with the simultaneous establishment of a specialized network of reference laboratories at children's oncohematology centers throughout the country.

In the ALL IC 2009 protocol, MRD by flow cytometry was analyzed. This method is available in most countries. Flow cytometry is less sensitive (10^−3^–10^−4^) than PCR-MRD, but the advantages are execution speed, availability and economic feasibility. The monoclonal antibody panels used in MRD monitoring were designed from the initial immunophenotype of each case by selecting markers conjugated to an eight-color immunostaining panel (EuroFlow 8-color antibody panel). MRD was evaluated in bone marrow samples on day 15 of the induction phase with a sensitivity of 0.01% or better. According to the ALL IC 2009 protocol, patients classified as SR should have FCM-MRD < 0.1%, patients with FCM-MRD > 0.1 and < 10% will be upgraded to IR, and those classified as SR with > 10% will be included in the HR group. Patients classified as IR with FCM-MRD > 10% will be included in the HR group, and all others will remain as IR.

### Definition of central nervous system (CNS) status

CNS status 1 was defined as no clinical and imaging evidence of CNS disease and no blasts on cytospin of cerebrospinal fluid (CSF). CNS status 2 was defined as pleocytosis ≤ 5/µl with clearly identified blasts on cytospin of blood-contaminated CSF. CNS status 3 was defined as nontraumatic lumbar puncture with pleocytosis > 5/µl, a mass lesion in imaging studies of the brain and/or meninges or cranial nerve palsy unrelated to other origin, even if the CSF is blast-free.

### Treatment and supportive care

The ALL IC-BFM 2002 and 2009 protocols were randomized trials of the I-BFM-SG (International Berlin-Frankfurt-Munster Study Group) for the therapy of childhood acute lymphoblastic leukemia, excluding Burkitt’s leukemia. According to the ALL IC 2002 and ALL IC 2009 protocols, children were stratified into three risk groups: standard (SRG), intermediate (IRG) and high (HRG). The stratification criteria of both protocols are described in Table 2. Patients treated according to these protocols received induction of remission, consolidation, reinduction and maintenance therapy. IRG patients with B-cell precursor ALL treated according to the ALL IC 2002 protocol received methotrexate (MTX) 2 g/m^2^, and patients with T-ALL received MTX 5 g/m^2^. IRG children treated according to the ALL IC 2009 protocol were administered MTX 5 g/m^2^ by modification of the Polish Pediatric Leukemia and Lymphoma Study Group. HR patients received early intensification (Protocol IB Augmented) according to the ALL IC 2009 regimen. These patients with HLA-matched related donors qualified for stem cell transplantation (SCT) after the third HR course in both protocols. Details of ALL IC-BFM 2002 therapy were published by Stary et al.^20^. Supportive therapy was used in accordance with the standards of Polish centers and recommendations of the therapeutic protocols.

**Table 2:** Stratification to risk group according with the ALL IC 2002 and ALL IC 2009 protocols.

	ALL IC 2002	ALL IC 2009
SRG	peripheral blood: blast < 1000/µl on day 8 and age ≥ 1 year to < 6 years and WBC < 20,000/µl and M1/M2 marrow on day 15and M1 marrow on day 33	peripheral blood: blast < 1000/µl on 8 day and age ≥ 1 year to < 6 years and WBC < 20 000/µl and **FC MRD < 0.1% on day 15** or M1/M2 marrow on day 15 and M1 marrow on day 33
IRG	Peripheral blood: blast < 1000/µl on day 8 and age < 1 year or ≥ 6 years and/or WBC > 20 000/µl and M1/M2 marrow on day 15 and M1 marrow on day 33 or criteria for SRG but marrow M3 on day 15 and M1 on day 33	Peripheral blood: blast < 1000/µl on day 8 and age < 1 year or ≥ 6 years and/or WBC > 20,000/µl and **FC MRD > 0.1% < 10% on day 15** or M1/M2 marrow on day 15 and M1 marrow on day 33
HRG	Criteria for IRG and M3 marrow on day 33 or in peripheral blood blasts ≥ 1000/µl or M2/M3 marrow on day 33 or translocation t (9;22)*BCR/ABL1* or t(4;11)*KMT2A/AFF1*(*formerly MLL-AF4*)	Criteria for IRG and **FC MRD > 10% on day 15** or criteria for IRG and M3 marrow on day 15 or criteria for SRG and **FC MRD > 10% on day 15** or translocation t (9;22)*BCR/ABL1* or t(4;11)*KMT2A/AFF1(formerly MLL-AF4)* or in peripheral blood blasts ≥ 1000/µl or marrow M2/M3 on day 33 or hypodiploidy ≤ 44

*SRG* standard risk group, *IRG* intermediate risk group, *HRG* high risk group, *WBC* white blood cell count, *FC-MRD* flow cytometry minimal residual disease, Bone marrow: M1: blast < 5%, M2: blast ≥ 5% < 25%, M3: blast ≥ 25%.

### Statistical analysis

Nominal variables are presented as n (% of group), with age presented as the mean ± SD. Data normality was verified with the Shapiro–Wilk test as well as a visual assessment of histograms. Groups were compared with Fisher’s exact test, the χ^2^ test or t-test, as appropriate. Due to the large group size, the effect size was calculated to assess the clinical importance of statistically significant differences: Cohen’s d for continuous variables and Cramer-V for nominal variables. OS and EFS curves were prepared with the Kaplan–Meier survival analysis method, including 95% confidence intervals. The log-rank Cox–Mantel test was used to compare the OS and EFS rates between subgroups. Cumulative death and relapse risk curves were estimated based on the Kaplan–Meier method with 95% confidence intervals. All tests were two-tailed with a level of significance of *P* = 0.05. Analysis was conducted in R software, version 3.5.2.
